# Supplementary material for: Linking spontaneous and stimulated spine dynamics
Source: Commun Biol. 2023 Sep 11;6:930. doi: 10.1038/s42003-023-05303-1 (PMC10495434; doi:10.1038/s42003-023-05303-1)
Supplement: Supplementary file 2 — Supplementary material [file 42003_2023_5303_MOESM2_ESM.pdf]

Supplementary Information

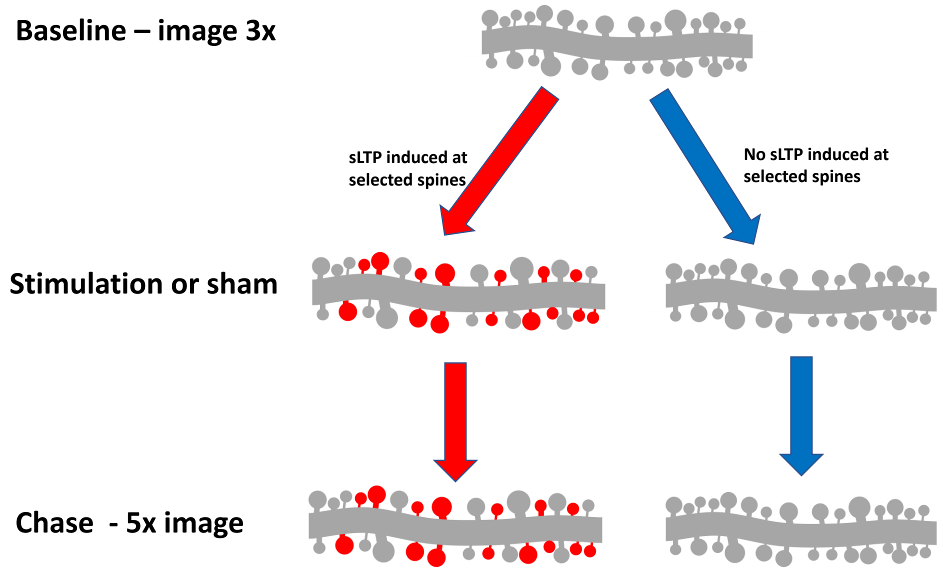

Supplementary Figure 1: **Illustration of the stimulation and sham experiments to study the activity-dependent and activity-independent plasticity, respectively.** Under both paradigms, the initial three time points are used as a pre-stimulation baseline. For the stimulation scenario we select a set of spines for glutamate uncaging to induce LTP between the 3rd and 4th time point. In the sham paradigm, these spines are similarly selected and receive pulses of the uncaging laser, but in the absence of the caged glutamate. The next 5 time points are then used to study the evolution of the spines within the image.

Supplementary Note 1: Links to biological mechanisms

The model presented in this work can also be viewed through a molecular lens, heavily inspired by Shomar et al. [42], although we will restrict ourselves from making definitive statements. First, let us consider the long-term stochastic component. Consider a spine of a given size (quantified in this study by area, see Methods); the number of molecules in the spine's wall determines this size. Some of these molecules can leave the wall at each time step with some probability. Simultaneously, free-floating molecules can enter the spine and bind with some probability. The larger spines will naturally be more variable because more molecules in the wall can escape. We note that the average large spine tends to shrink in size, which implies that the number of molecules that detach from the spine wall is larger than the number of free molecules that bind to it. Therefore, we propose that the probability of the unbinding must be larger than the binding probability. However, we also note that small spines tend to grow, meaning that, in this case, more binding must occur. We note that the size of the spine (i.e., the number of molecules in the wall) may affect the binding/unbinding probabilities. However, to avoid such a complex relationship, we propose that there is a relatively fixed amount of molecules that can bind to a spine. This would then lead to, on average, more binding than unbinding for small spines, while we would have the opposite for large spines. This neuron-specific homeostatic mechanism then leads to increased energy efficiency (due to the degradation of expensive-to-maintain large spines), which may have implications when scaled up to the neuronal level.

Turning to the mean-reverting portion of the model, we have two key factors: the *drift* and the *negative momentum*. Beginning with the drift, we note that this quantity requires a constant parameter $\bar{\mu}$, which is constant across all spine sizes.

Finally, the negative momentum term can be interpreted using the following mechanisms related to the actin dynamics inside the spines. Several studies[30,31,58,59] have investigated the mechanical properties of actin networks and showed that

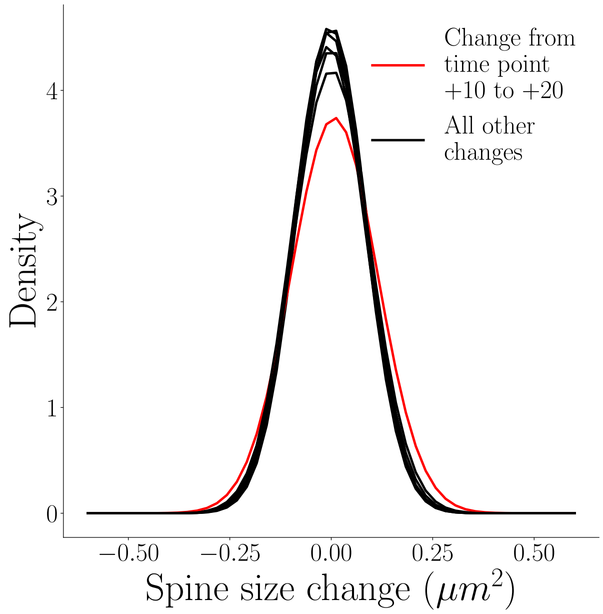

Supplementary Figure 2: **Alternative depiction of the spine changes distributions (which display Gaussian features) from Fig. 1d.** The distribution of spine size changes of activity-independent plasticity do not differ from each other significantly (K.S. test), apart from the single change distribution from time point +10 to +20, which is marked with a red line.

these cytoskeletal structures can exhibit wave-like oscillations in time with a period of $1 - 100$ s. This behavior emerges from a complex interaction between the actin filament rigidity, the spine membrane surface tension, and the stochastic polymerization-depolymerization processes. We include the negative-momentum term in our model to incorporate the actin-mediated negative correlation in the spine changes we observe in the data. This term can be considered an abstract counterpart of the intricate actin interaction network.

These spontaneous spine oscillations potentially prevent a "winner-takes-all" effect which could lead to uncontrolled growth/shrinkage. A possible functional implication is that the trend to reverse previous changes serves to suppress progressive spine size changes that are locked in one direction. The stimulation induced forces that allow for a collective shift of the spines to larger sizes. Additionally, it is plausible that these oscillations provide the spines with a "set point" within biologically plausible bounds by pushing the spine sizes from both edges toward the middle. Therefore, given that natural random protein movement within the spines will lead to spine changes, these oscillations could provide a mechanism to help preserve a stable synaptic size average and thereby contribute to network stability.

Interestingly, the experimental conditions that give rise to these negative spine correlations are yet to be fully understood. For example, Minerbi et al. [13] did not report a negative correlation when studying long-term random fluctuations in dissociated cortical cultures at 30-minute intervals. On the other hand, Yasumatsu et al. [12] reported a slight negative correlation (-0.189) when considering the change in hippocampal slice cultures over a period of days. From these and our results, two avenues of study arise. On the one hand, understanding the exact temporal relation between the oscillations and the size of the experimentally considered time bins would provide insight into whether the negative momentum and its amplitude does indeed preserve not only average synaptic size but helps maintain stable network and information retention properties. On the other hand, understanding the pharmacological conditions and cell environment controlling or inhibiting these oscillations e.g. [11,24] could provide insights into the mechanisms giving rise to or preventing negative correlations in synaptic size changes.

Interestingly, we observed that plasticity induction maintained the negative momentum term in the spine dynamics. On the one hand, the stimulation altered the probabilities of binding/unbinding of molecules in the spines, which affected the distribution of spine changes. On the other hand, the stimulation protocol lead to a new stable molecular configuration that established a new $\bar{\mu}$ in the drift component.

Finally, the presented log-normal model can be conceptually linked to the process known as geometric Brownian motion (GBM) (also known as exponential Brownian motion). By simulating the spine sizes as individual GBM particles, we could feasibly reproduce the results of the experimental dataset. For instance, similarly to how an ensemble of Brownian motion

particles will generate a Gaussian distribution, an ensemble of GBM particles will generate a log-normal distribution [60], as seen in our stable distribution of sizes. Furthermore, in this framework, future states (sizes) are defined by the stochastic differential equation for state S_t

$$dS_t = \mu S_t \, dt + \sigma S_t \, dW_t \tag{17}$$

where dW_t represents standard Brownian motion, μ, the relative drift and σ, the relative volatility. The dependence of the change on the current state, S_t, mirrors the effect that larger spines are more variable than smaller spines. Previous work in this direction includes the study by Yasumatsu et al. [12] and Hazan and Ziv [24], the later has shown that the model Eq. 17 is interchangeable under certain conditions with the Kesten process. However, adequately estimating the parameters underlying GBM (μ and σ) is non-trivial and, due to our low resolution in time, not feasible in this study.

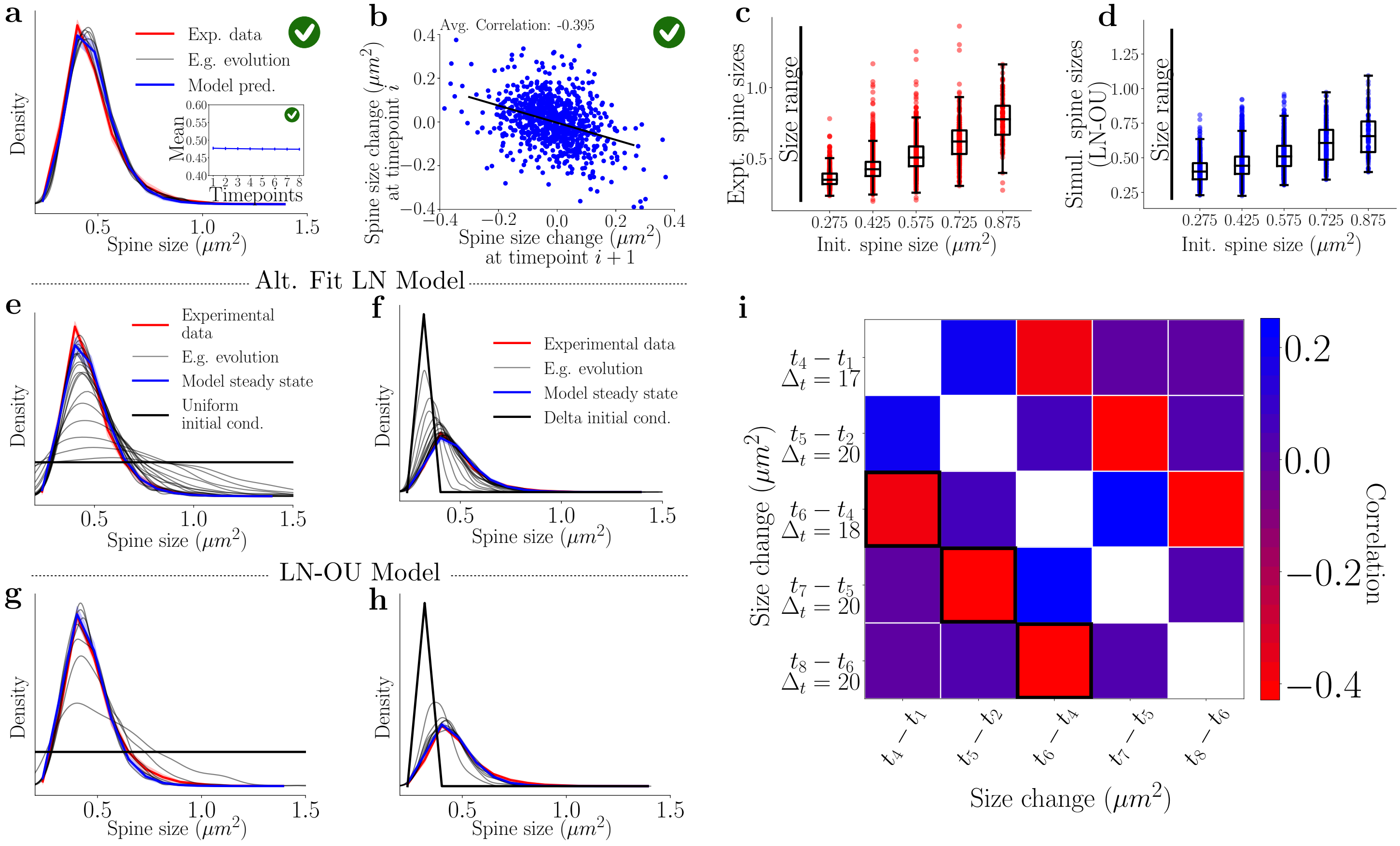

Supplementary Figure 3: **By adding the negative momentum term to the Alt. Fit LN model (see equation (9)) reproduces all aspects of the experimental data without a drift component. Additionally, throughout the experiment, a wide range of spine sizes are observed that can be reproduced by both log-normal models (Alt. Fit LN and LN-OU, equation (4)). a)** The distribution obtained using the altered linear fits (Fig. 2e) in the Alt. Fit model with the negative momentum term (equation (9)). Significant stability is observed (the inset represents the mean of the simulations). "E.g. evolution" refers to one example simulation of spine sizes. **b)** The correlation of this model demonstrates significantly more negative correlation in comparison to the Alt. Fit model without the negative momentum (cf. Fig. 2g), in line with the desired model goals. **c)** By splitting the spines into categories based on their initial sizes, we can characterize all the subsequent sizes of that initial size. Significant intermixing is observed. The left line represents the total set of spine possible spine sizes. **d)** The previous categories can be simulated using the LN-OU model. We observe remarkable similarity in the size distributions as seen in c). **e)-f)** To verify that the Alt. Fit LN Model satisfies our modeling goal 3 (generating the experimental stable distribution after starting at a different one), we initialized all spines sizes to be uniformly represented or all spine sizes with one value (= 0.25 μm^2) (black lines in e) and f), respectively). We then use this initial state and simulate the next distributions using the Alt. Fit Model. We note that the log-normal model requires a large amount of steps to reach the steady state. **g)-h)** Similarly, we verify that the LN-OU Model satisfies modeling goal 3. In both cases (initial uniform distribution and initial delta distribution), the LN-OU requires fewer steps to achieve the desired steady state. **i)** In contrast to Fig. 1g, where consecutive size changes were compared, here we find all time point differences that are ≈ 20 minutes and compare these against each other. We see that points immediately following each other (highlighted by black squares) are negatively correlated even over this extended time period.

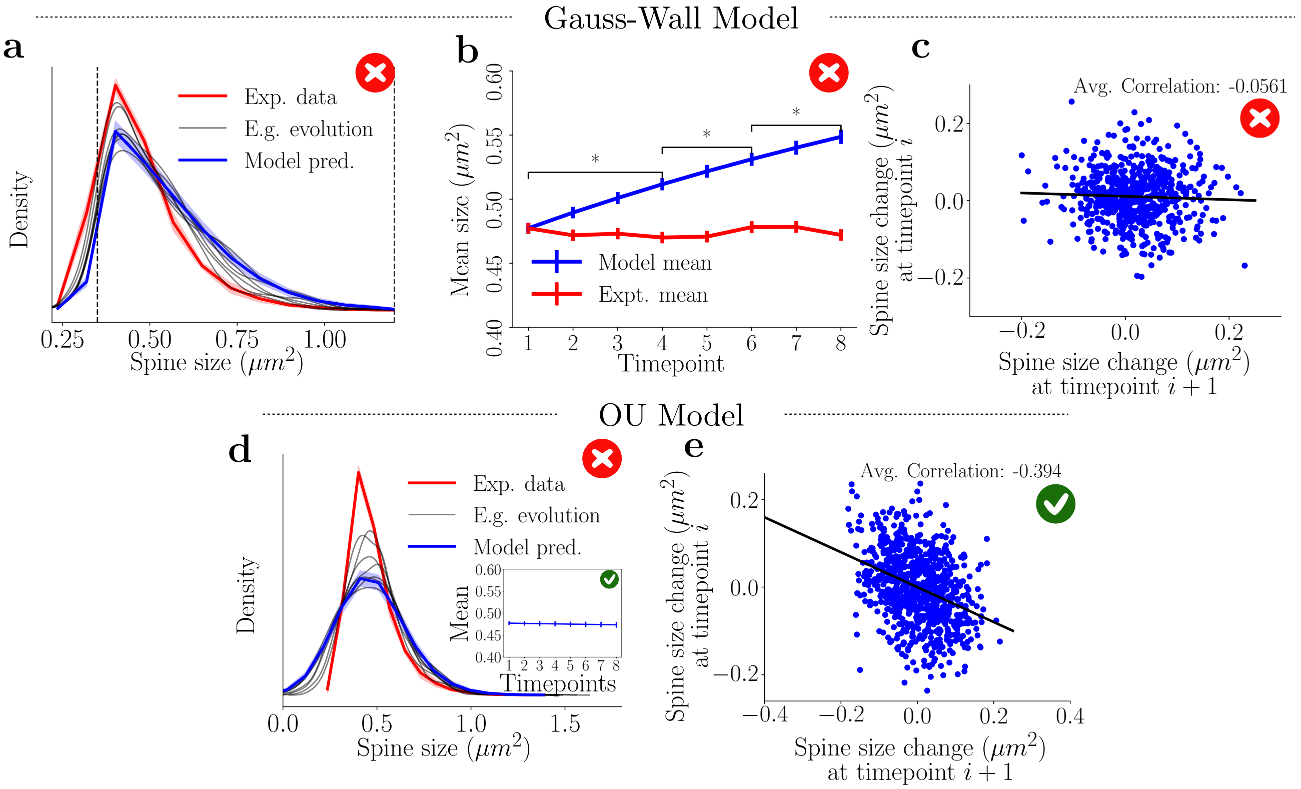

Supplementary Figure 4: **Evaluating model performance of models based on the Gaussian process.** We will mark with a red cross model features that violate an experimental observation, while the green tick will denote a model-data match. **a)** Starting with a Gauss wall model (equation (7)) where μ and σ were obtained from experimental data (Fig. 1e) we find that the stability and shape of the experimentally recorded spine is not well captured by the model. *E.g. evolution* refers to one example simulation of spine sizes. **b)** The mean of the Gaussian wall model, exhibits a significant increase over time (blue) while the data shows a time-stable mean (red). $*$ refers to $p < 0.05$ of a two-sided t-test comparing the two timepoints at the endpoints of the horizontal bar. **c)** The Gaussian wall model cannot capture the negative correlations observed in the data (see Fig. 1g). **d)** The spine size distribution in the OU model lacks temporal stability but exhibits a stable mean (inset). **e)** The OU model can capture the temporal correlations observed in the experimental data.

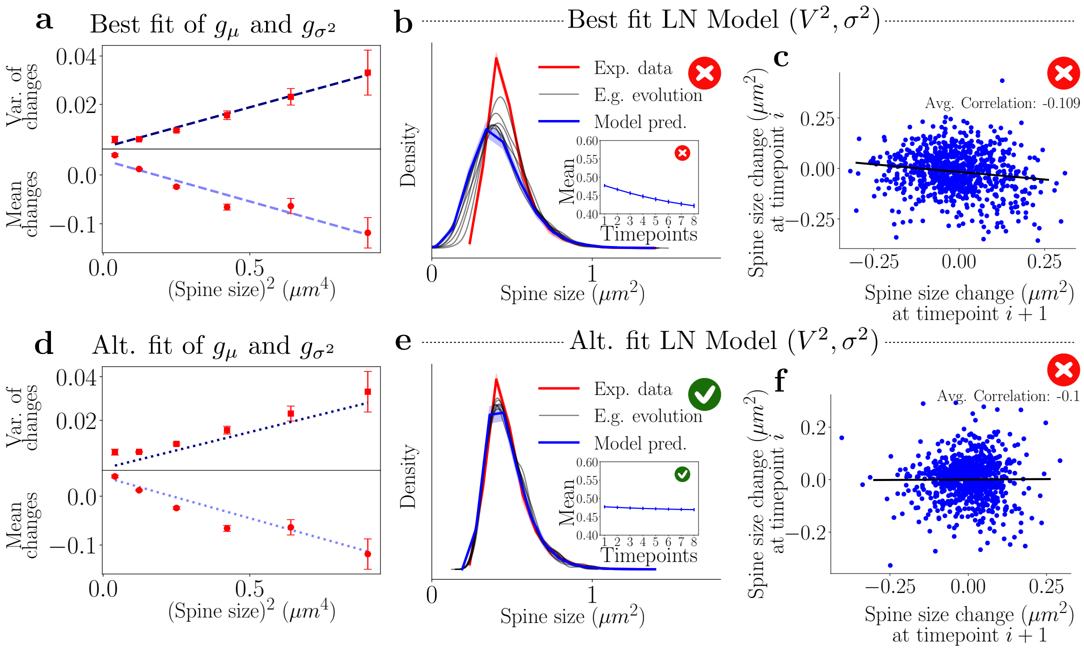

Supplementary Figure 5: **Linear relations can be found between the variance (σ^2) and mean (μ) and the square of the spine sizes (V^2). a)** Linear relations between the square of the spine sizes and sample change means, g_μ, and variances, g_{σ^2}, of activity-independent plasticity show good agreement. **b)** Similar to the earlier fits in Fig. 2, simulations using the linear fits from **a)** do not result in a stable distribution. The inset represents the simulated mean, which decreases significantly. **c)** The correlation measured across one step of the best fit log-normal simulations. The slope is ≈ 0.1, which is smaller than required for reproducing the experimental data. **d)** Altered linear fits for the mean and variance are used to achieve modeling goals. **e)** Distribution obtained from the simulation when the altered linear fits of the sample mean and standard deviation are used. The stability of the distribution is achieved as well as that of the mean (inset). **f)** The correlation obtained from one step of the Alt. Fit LN model simulations. The slope is ≈ 0.1, which is smaller than required for reproducing the experimental data.

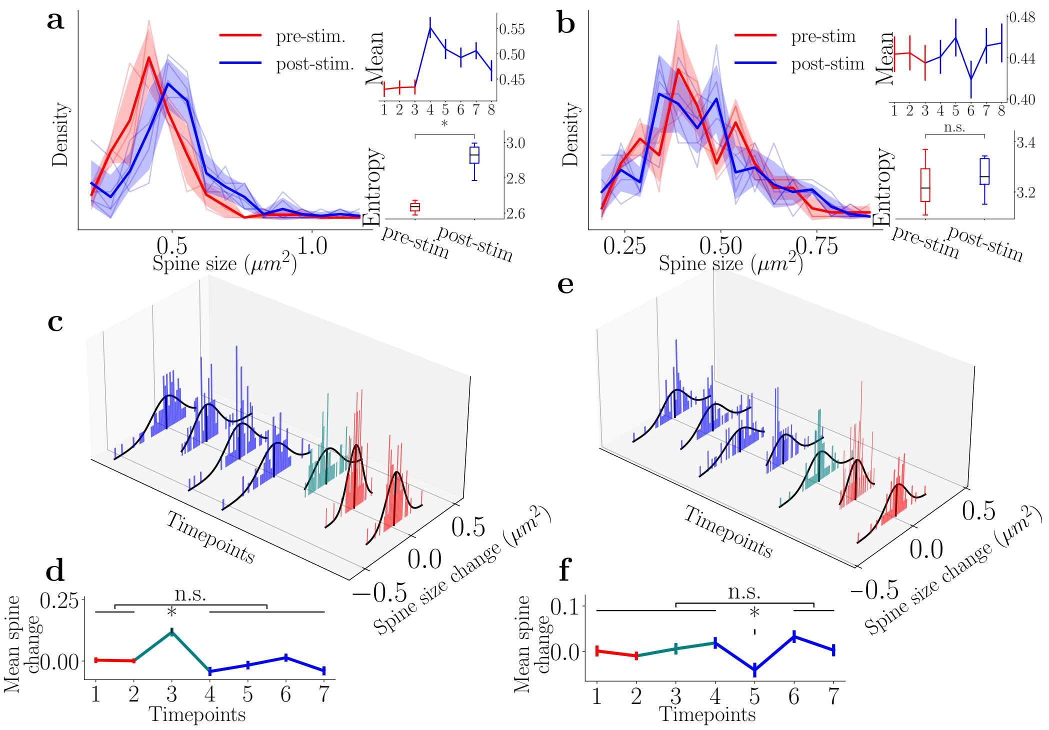

Supplementary Figure 6: **Similar to the simultaneous stimulation of 15 spines, 7 spines LTP induction leads to a distinctly shifted distribution. a)** Distribution of spine sizes for the homosynaptic spines for different time points, with red and blue referring to pre- and post-stimulation, respectively. Sample mean and entropy are also shown. **b)** Distribution of spine sizes for the heterosynaptic spines (within 4 μm of any stimulation site) for different time points, with red and blue referring to pre- and post-stimulation, respectively. Sample mean and entropy are also shown. **c)** Distributions of the homosynaptic spines changing from time point to time point follow a Gaussian distribution (even the stimulation time point) that is shifted towards positive values. Teal represents the change directly after the stimulation. **d)** The mean of spine change from time point to time point of all of the homosynaptic spines. A one-way ANOVA test reveals that only the stimulation time point has a significantly different spine size change. All other time points cannot be significantly differentiated from activity-independent plasticity. **e)** Distributions of the heterosynaptic spines changing from time point to time point follow a Gaussian distribution (even the stimulation time point). Teal represents the change directly after the stimulation. **f)** The mean of spine change from time point to time point of all of the heterosynaptic spines. A one-way ANOVA test reveals that all means (except for the 4th time point) are of the same distribution and so cannot be significantly differentiated from activity-independent plasticity.
