# Supplementary material for: Linking spontaneous and stimulated spine dynamics
Source: Commun Biol. 2023 Sep 11;6:930. doi: 10.1038/s42003-023-05303-1 (PMC10495434; doi:10.1038/s42003-023-05303-1)
Supplement: Supplementary file 3 — Reporting Summary [file 42003_2023_5303_MOESM3_ESM.pdf]

nature portfolio

Corresponding author(s): Tatjana Tchumatchenko

Last updated by author(s): Aug 17, 2023

Reporting Summary

Nature Portfolio wishes to improve the reproducibility of the work that we publish. This form provides structure for consistency and transparency in reporting. For further information on Nature Portfolio policies, see our Editorial Policies and the Editorial Policy Checklist.

Statistics

For all statistical analyses, confirm that the following items are present in the figure legend, table legend, main text, or Methods section.

n/a	Confirmed	
☐	☒	The exact sample size (*n*) for each experimental group/condition, given as a discrete number and unit of measurement
☒	☐	A statement on whether measurements were taken from distinct samples or whether the same sample was measured repeatedly
☐	☒	The statistical test(s) used AND whether they are one- or two-sided<br>*Only common tests should be described solely by name; describe more complex techniques in the Methods section.*
☐	☒	A description of all covariates tested
☐	☒	A description of any assumptions or corrections, such as tests of normality and adjustment for multiple comparisons
☐	☒	A full description of the statistical parameters including central tendency (e.g. means) or other basic estimates (e.g. regression coefficient) AND variation (e.g. standard deviation) or associated estimates of uncertainty (e.g. confidence intervals)
☐	☒	For null hypothesis testing, the test statistic (e.g. *F*, *t*, *r*) with confidence intervals, effect sizes, degrees of freedom and *P* value noted<br>*Give P values as exact values whenever suitable.*
☒	☐	For Bayesian analysis, information on the choice of priors and Markov chain Monte Carlo settings
☒	☐	For hierarchical and complex designs, identification of the appropriate level for tests and full reporting of outcomes
☐	☒	Estimates of effect sizes (e.g. Cohen's *d*, Pearson's *r*), indicating how they were calculated

Our web collection on statistics for biologists contains articles on many of the points above.

Software and code

Policy information about availability of computer code

Data collection	An in-house python code was used, which can be found at: https://github.com/meggl23/MultiSpinePlasticity. Briefly, it relies on semi-automated algorithm to generate synapse ROIs, which are then used to study the temporal dynamics via the area of those ROIs.
Data analysis	An in-house python code was used for analysis (jupyter-notebook) that relied on algorithms from scipy and numpy for the data analysis. This code can be found at https://github.com/meggl23/SpontaneousSpines

For manuscripts utilizing custom algorithms or software that are central to the research but not yet described in published literature, software must be made available to editors and reviewers. We strongly encourage code deposition in a community repository (e.g. GitHub). See the Nature Portfolio guidelines for submitting code & software for further information.

Data

Policy information about availability of data

All manuscripts must include a data availability statement. This statement should provide the following information, where applicable:

- Accession codes, unique identifiers, or web links for publicly available datasets
- A description of any restrictions on data availability
- For clinical datasets or third party data, please ensure that the statement adheres to our policy

Experimental data sets included in the manuscript and the code to generate the figures can be found in the following public github repository: github.com/meggl23/SpontaneousSpines with DOI: 10.5281/zenodo.7885342.

Research involving human participants, their data, or biological material

Policy information about studies with human participants or human data. See also policy information about sex, gender (identity/presentation), and sexual orientation and race, ethnicity and racism.

Reporting on sex and gender	N/A
Reporting on race, ethnicity, or other socially relevant groupings	N/A
Population characteristics	N/A
Recruitment	N/A
Ethics oversight	N/A

Note that full information on the approval of the study protocol must also be provided in the manuscript.

Field-specific reporting

Please select the one below that is the best fit for your research. If you are not sure, read the appropriate sections before making your selection.

☒ Life sciences ☐ Behavioural & social sciences ☐ Ecological, evolutionary & environmental sciences

For a reference copy of the document with all sections, see nature.com/documents/nr-reporting-summary-flat.pdf

Life sciences study design

All studies must disclose on these points even when the disclosure is negative.

Sample size	The sample size of spines was determined by studying the maximum intensity projection of each hippocampal slice, and any spine that could not be clearly identified at every time point was not included in the analysis. This lead to 830 spines for the activity-independent data set, and 338 spines, of which 187 were directly stimulated, for the data set with 15 simultaneous stimulations.
Data exclusions	No Data was excluded
Replication	Findings in this document replicates other findings seen in previous literature, albeit in slightly different conditions.<br><br>Additionally, as the full activity-independent dataset consisted of slightly different experiments, each individual experiment was analysed separately to ensure that the results were not dominated by the ensemble statistics. In each of the experiments no signficant differences were found, and results were replicated across the board.
Randomization	Experimental groups were defined by the experimental paradigm; activity-independent or activity-dependent. Due to this no randomization was required.
Blinding	Blinding is not relevant to this study, as it was not necessary for the purpose of the analysis.

Reporting for specific materials, systems and methods

We require information from authors about some types of materials, experimental systems and methods used in many studies. Here, indicate whether each material, system or method listed is relevant to your study. If you are not sure if a list item applies to your research, read the appropriate section before selecting a response.

Materials & experimental systems

n/a	Involved in the study	
☒	☐	Antibodies
☒	☐	Eukaryotic cell lines
☒	☐	Palaeontology and archaeology
☐	☒	Animals and other organisms
☒	☐	Clinical data
☒	☐	Dual use research of concern
☒	☐	Plants

Methods

n/a	Involved in the study	
☒	☐	ChIP-seq
☒	☐	Flow cytometry
☒	☐	MRI-based neuroimaging

Animals and other research organisms

Policy information about studies involving animals; ARRIVE guidelines recommended for reporting animal research, and Sex and Gender in Research

Laboratory animals	Organotypic hippocampal slices of postnatal day 6-7 Wistar rat pups were used in this study.
Wild animals	The study did not involve wild animals
Reporting on sex	Findings only apply to one sex
Field-collected samples	The study did not involve field-collected samples
Ethics oversight	All animal experiments were approved by the RIKEN Animal Experiments Committee and performed in accordance with the RIKEN rules and guidelines. Animal Experiment Plan Approval no. W2021-2-015(3)

Note that full information on the approval of the study protocol must also be provided in the manuscript.
